# The staged roll-out of an MRSA intervention bundle in Signapore featuring universal active surveillance

**DOI:** 10.1186/1753-6561-5-S6-O84

**Published:** 2011-06-29

**Authors:** D Fisher, R Lin, LY Hsu, O Kooi Li, TM Ng, P Tambyah

**Affiliations:** 1 National University Hospital, Singapore, Singapore

## Introduction / objectives

Between 2004 and 2006, Singapore’s 997 bed National University Hospital (NUH) had 62 hospital acquired MRSA bacteraemias/year. Another 200 inpatients/year had new non blood borne MRSA infections. Minimal active surveillance was undertaken.

## Methods

Between 2006 and 2010, universal active surveillance (on admission and discharge), isolation/cohorting, data feedback loops and standardised hand hygiene (HH) audits were rolled out in a stepwise fashion across NUH. A comprehensive hand hygiene programme was institutionalised. MRSA acquisition was defined as having a positive exit swab yet negative entry and no known previous MRSA. Ward specific HH compliance and acquisition rates were fed back via public displays on each ward monthly.

## Results

All adult medical and surgical wards had implemented the bundle by July 2010. In that month 3620 entry swabs were taken. Compliance rates with exit swabs are > 85%. MRSA acquisition fell from 10.1 to 3.1% and from 9.2 to 2.8% in our Intensive Care Units and general wards respectively. Nosocomial bacteraemia rates fell from 0.23 /1000 pt days in 2008 to 0.11 in 2010. The MRSA burden however remains high hospital wide and vancomycin use has not fallen.

## Conclusion

The value of active surveillance remains controversial and near impossible to prove in a real life setting in the short term. A major reservoir continues to exist at NUH. A high baseline transmission rate may represent the initial identification of the reservoir of MRSA using an imperfect screening tool. Genuine success can only be acknowledged with a fall in infection rates, the total burden plus antibiotic useage. Acquisition rates are a useful interim tool but healthy scepticism is required during the collection of early data during major MRSA prevention programmes.

## Disclosure of interest

None declared.

